# *In situ* ultra-small-angle X-ray scattering study under uniaxial stretching of colloidal crystals prepared by silica nanoparticles bearing hydrogen-bonding polymer grafts

**DOI:** 10.1107/S205225251600556X

**Published:** 2016-04-19

**Authors:** Ryohei Ishige, Gregory A. Williams, Yuji Higaki, Noboru Ohta, Masugu Sato, Atsushi Takahara, Zhibin Guan

**Affiliations:** a Institute for Materials Chemistry and Engineering (IMCE), Kyushu University, 744 Motooka, Nishi-ku, Fukuoka 819-0395, Japan; b Department of Chemistry, 1102 Natural Sciences 2, University of California, Irvine, CA 92697, USA; c Japan Synchrotron Radiation Research Institute (JASRI/SPring-8), Sayo-cho, Sayo-gun, Hyogo 679-5198, Japan

**Keywords:** polymer-grafted nanoparticles, colloidal crystals, uniaxial stretching, ultra small-angle X-ray scattering (USAXS)

## Abstract

A molded film of single-component polymer-grafted nanoparticles (SPNP), consisting of a spherical silica core and densely grafted polymer chains bearing hydrogen-bonding side groups capable of physical crosslinking, was investigated by *in situ* ultra-small-angle X-ray scattering (USAXS) measurement during a uniaxial stretching process. Static USAXS revealed that the molded SPNP formed a highly oriented twinned face-centered cubic (f.c.c.) lattice structure with the [11−1] plane aligned nearly parallel to the film surface in the initial state. Structural analysis of *in situ* USAXS using a model of uniaxial deformation induced by rearrangement of the nanoparticles revealed that the f.c.c. lattice was distorted in the stretching direction in proportion to the macroscopic strain until the strain reached 35%, and subsequently changed into other f.c.c. lattices with different orientations. The lattice distortion and structural transition behavior corresponded well to the elastic and plastic deformation regimes, respectively, observed in the stress–strain curve. The attractive interaction of the hydrogen bond is considered to form only at the top surface of the shell and then plays an effective role in cross-linking between nanoparticles. The rearrangement mechanism of the nanoparticles is well accounted for by a strong repulsive interaction between the densely grafted polymer shells of neighboring particles.

## Abstract 2

The change in the crystal structure of polymer-grafted nanoparticles during uniaxial stretching was investigated by simultaneous ultra-small-angle X-ray scattering and stress–strain measurement.

## 1. Introduction   

Colloidal crystal nanocomposites composed of spherical particles (Hachisu *et al.*, 1973 ▸; Kose & Hachisu, 1974 ▸; Pusey & van Megen, 1986 ▸; van Megen & Underwood, 1993 ▸; Okubo, 1993 ▸; Dinsmore *et al.*, 1998 ▸) are intriguing materials due to the capability of spontaneous formation of close-packed crystal structures. It is well known that repulsive interactions between particles are the driving force of the crystallization process of colloidal crystals (Alder & Wainwright, 1957 ▸; Hoover, 1968 ▸; Alder, 1968 ▸). Colloidal crystals are classified into two categories according to the type of repulsive force: one is a *hard* colloid (Hachisu *et al.*, 1973 ▸; Kose & Hachisu, 1974 ▸; Pusey & van Megen, 1986 ▸; van Megen & Underwood, 1993 ▸), where a contact force derived from the hard-sphere potential (excluded-volume interaction) dominates the repulsive force, and the other is a *soft* colloid (Okubo, 1993 ▸; Dinsmore *et al.*, 1998 ▸), where a non-contact force, *e.g.* osmotic forces, electro­static forces between charged particles *etc.*, dominates the repulsive force (Witten & Pincus, 1986 ▸, 2010 ▸; Okubo, 1993 ▸; Hachisu *et al.*, 1973 ▸). Polymer-grafted nanoparticles, consisting of a hard spherical core and a shell of densely grafted polymer chains, are called *semi-soft* colloids because the particles have a character intermediate between those of hard and soft colloids (Ohno *et al.*, 2006 ▸, 2007 ▸; Morinaga *et al.*, 2008 ▸). In semi-soft colloidal systems, both the hard-sphere potential of the core and the osmotic force induced by the highly concentrated shell of grafted polymer on the core contribute to the repulsive force. The close-packed structures of semi-soft colloids also present a character intermediate between those of hard and soft colloids (Ohno *et al.*, 2007 ▸).

Compared with other colloidal crystals, polymer-grafted nanoparticles have enhanced processability even in the bulk state, because the grafted-polymer shell is expected to add fluidity and toughness without solvent (Kolmakov *et al.*, 2009 ▸; Iyer *et al.*, 2015 ▸), while traditional hard and soft colloidal crystals in suspension systems are usually fragile and difficult to use in the bulk state, unless the crystal structure is fixed by a gelation reaction of the matrix (Toyotama *et al.*, 2005 ▸; Huang *et al.*, 2014 ▸). Previously, we synthesized melt-processable spherical silica particles with flexible (low *T*_g_) and ‘sticky’ hydrogen-bonding polymers grafted onto the surface, which formed structurally colored films (Williams *et al.*, 2015 ▸). These liquid-less colloidal systems of polymer-grafted nanoparticles are called single-component polymer-grafted nanoparticles (SPNPs). The nanoparticles form a face-centered cubic (f.c.c.) crystal with a small grain size (or random hexagonal close-packed structure, r.h.c.p.) in the molded film, as established by ultra-small-angle X-ray scattering (USAXS) measurements; it should be noted that f.c.c. twinned crystals having a small grain-size and/or second-order disorder are difficult to distinguish from an r.h.c.p. crystal (Loose & Ackerson, 1994 ▸; Daniel *et al.*, 2000 ▸). In these SPNPs, the hydrogen bond enables the matrix polymer to crosslink physically and provides robust mechanical properties such as toughness, elasticity, self-healing and so on. Therefore, the SPNP films were not only able to be molded in the bulk state at high temperature (100°C), but could also be deformed at ambient temperature by uniaxial stretching, and the structural color (Bragg reflection of visible light) was changed controllably by mechanical strain.

Numerous studies of the crystal structure of spherical colloids have been conducted based on USAXS (Förster *et al.*, 2011 ▸; Vos *et al.*, 1997 ▸; Petukhov *et al.*, 2001 ▸; Abramova *et al.*, 2009 ▸), light-scattering methods (Pusey *et al.*, 1989 ▸; Ackerson, 1990 ▸), transmission electron microscopy (Sakamoto *et al.*, 2002 ▸) and confocal fluorescence microscopy (van Blaaderen & Wiltzius, 1995 ▸; Gasser *et al.*, 2001 ▸). Of these methods, USAXS has some notable advantages, including the capability of *in situ* measurement of opaque samples, measurement of neat samples without fluorescent dye, quantitative results *etc*. In this study, the crystal deformation of a cross-linked colloidal crystal under strain was investigated and the mechanism for the change in structural color under strain is discussed based on the crystal structure. The mechanical deformation process of the oriented colloidal crystal film of molded SPNPs was investigated by simultaneous *in situ* USAXS and stress–strain measurements during a uniaxial stretching process. The USAXS patterns were analyzed by comparing the observed diffraction points with those calculated by a rearrangement model of the nanoparticles. The relationship between the mechanical properties and the local structure (lattice structure) is discussed based on the stress–strain curve (SS-curve) and the colloidal crystal structure derived from USAXS analysis. Finally, the effect of the densely grafted polymer layer (condensed polymer brush layer) on the deformation mechanism is discussed.

## 2. Experimental   

**Figure 1:**
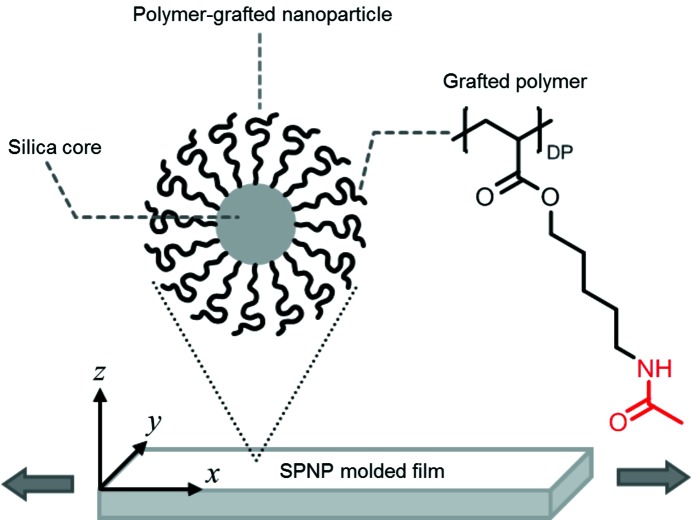
An illustration of the polymer-grafted nanoparticles, the SPNP molded thin film and the chemical structure of the grafted polymer. The *x*, *y* and *z* axes in Cartesian coordinates are defined as the longer direction, the width direction and the thickness direction of the film, respectively. The films are stretched uniaxially along the *x* axis, indicated by the large arrows. The X-ray beam is irradiated through the *z* and *y* directions, perpendicular to the through and edge planes, respectively.

The details of the synthetic procedure of the SPNPs and the preparation method of the molded film were reported previously (Williams *et al.*, 2015 ▸). The synthetic procedure of the SPNPs is briefly summarized. The diameter of the silica core of the SPNPs was 185 ± 5 nm. Initiators for surface-initiated atom-transfer radical polymerization (SI-ATRP) were fixed on the surface of the neat silica core. A hydrogen-bonding polymer containing an amide side group and poly(acrylate) backbone, poly(5-acetylaminopentyl acrylate), was grafted onto the silica core by SI-ATRP (see Fig. 1 ▸). The degree of polymerization of the grafted polymer was 310. The weight fraction of the grafted polymer was evaluated at 68 wt% by thermogravimetric analysis (TGA). The graft density of the polymer chain on the surface of the silica core was evaluated at 1.2 chains nm^−2^. The molded thin films were used for USAXS measurements, which were conducted on the BL19B2 and BL40B2 beamlines at SPring-8 (Japan Synchrotron Radiation Research Institute, Hyogo, Japan). The wavelengths of X-rays and the camera lengths were 0.0688 nm and 41.800 m, respectively, for BL19B2, and 0.150 nm and 4.240 m, respectively, for BL40B2. The camera lengths were calibrated using the diffraction of collagen fiber. In order to identify the orientation of the crystal structure, the X-ray beam was irradiated through the film in two different directions, one perpendicular to the *xy* (through) plane and the other perpendicular to the *xz* (edge) plane. Herein, the *x*, *y* and *z* axes of the Cartesian coordinates were defined as the directions parallel to the longest direction, the width direction and the thickness direction of the films, respectively. A schematic representation of the sample geometry is presented in Fig. 1 ▸. The films were stretched uniaxially at 10% min^−1^ along the *x* axis. Diffraction patterns were taken at preset intervals during the stretching process using a PILATUS-2M detector on BL19B2 and an ADSC Quantum 4R CCD camera on BL40B2. The exposure time per frame was 9 s for the PILATUS-2M detector and 0.05 s for the CCD detector. A tension tester, model OZ501 (Sentech, Oosaka, Japan), was used for the uniaxial stretching and the stress–strain measurements.

## 3. Results and discussion   

### 3.1. Crystal orientation in molded film and deformation model in uniaxial stretching process   

**Figure 2:**
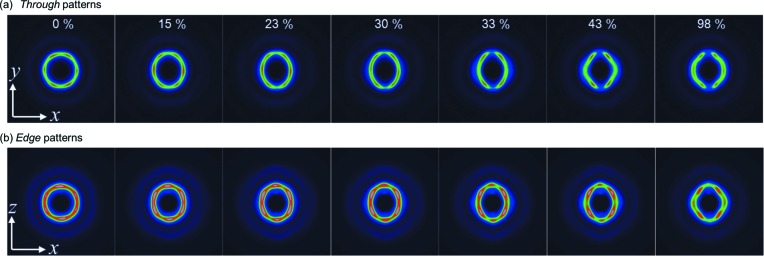
Typical USAXS patterns of the molded film during uniaxial stretching. (*a*) The through patterns (*yx* plane) and (*b*) the edge patterns (*xz* plane). The stretching rate was 10% min^−1^ in the *x* direction. The strain values are indicated in the upper patterns.

Typical USAXS patterns taken with X-rays irradiated perpendicular to the through and edge planes (the *xy* and *xz* planes, respectively) are shown in Fig. 2 ▸ (the strain values of the patterns are 0, 15, 23, 30, 33, 43 and 98%). At 0% strain, hexagonally symmetric diffractions were observed in both the through and edge patterns. In the through pattern, two diffractions, corresponding to the corners of the hexagon, appeared on the equator, while in the edge pattern the diffractions appeared on the meridian. These diffraction geometries are consistent with those from a twinned f.c.c. crystal, where the 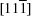 planes (one of the close-packed layers) are aligned parallel to the through plane (shear direction) (Versmold, 2010 ▸).

**Figure 3:**
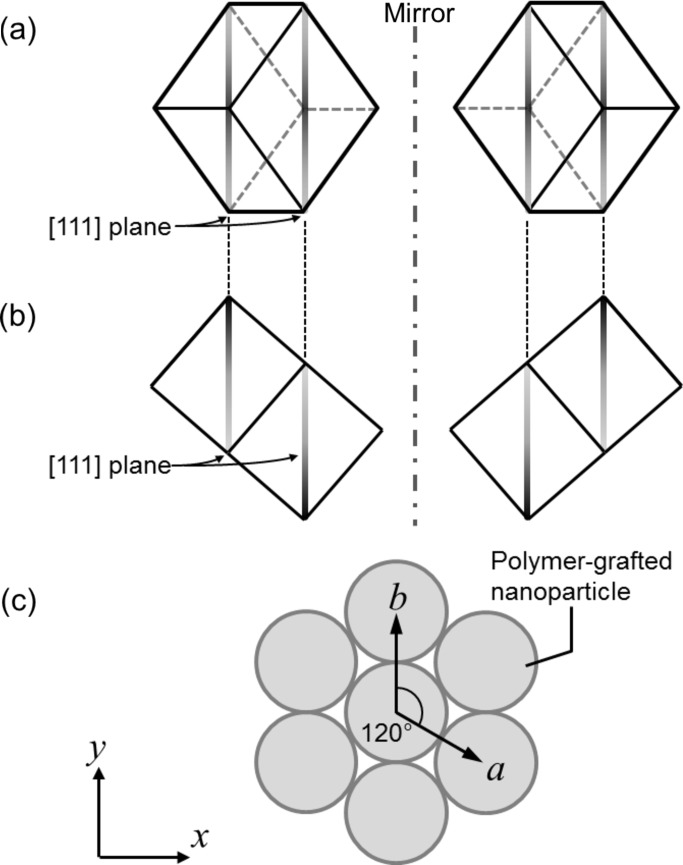
Schematic drawings of the f.c.c. lattices in the twinned crystal. (*a*) The view through the *z* axis (through view). (*b*) The view through the *y* axis (edge view). (*c*) The two-dimensional hexagonal lattice on the close-packed layer in the *xy* plane (through plane).

In twinned f.c.c. crystals, two grains coexist which are mirror images of each other and the [111] planes are shared at the interface of the two grains, as illustrated in Figs. 3 ▸(*a*) and 3 ▸(*b*). Herein, a two-dimensional hexagonal lattice of particles in the close-packed layer in the through plane is considered, and the lattice vectors are defined as **a** and **b** (Fig. 3 ▸ *c*). In the molded film, **b** is parallel to the *y* axis and perpendicular to the stretching direction (*x* axis). In this orientation, uniaxial mechanical stress is added in the direction perpendicular to the *b* axis (*y* axis). During the stretching process, all the diffractions approached the meridional line and the hexagonal symmetries of the diffractions were broken with increasing strain in the early stage, from 0 to 35% strain. Two deformation models may be considered for this stretching mode, where one of the close-packed layers is parallel to the stretching direction. For instance, one model is that the close-packed layers mutually slip, keeping the two-dimensional hexagonal arrangement (Kanai *et al.*, 2005 ▸; Amos *et al.*, 2000 ▸; Hamer *et al.*, 2014 ▸), and another is that the hexagonal close-packed arrangement is broken and the particles in the layer are separated in the stretching direction. In the present result, the distorted hexagonal symmetry of the diffractions indicates the latter deformation mechanism. In this regard, there have been few experimental results on the deformation of f.c.c. lattices of colloidal spheres under mechanical strain. Viel and co-workers reported cross-linked colloidal f.c.c. crystals (opal elastomer) consisting of nanoparticles with a poly(styrene) core and poly(ethyl acrylate) shell, and proposed a highly suggestive model for the deformation mechanism of the f.c.c. lattice during uniaxial stretching based on transmission electron micrograph observation results (Ruhl *et al.*, 2003 ▸; Viel *et al.*, 2007 ▸), although the stretching direction of the colloidal crystal in their work was different from our case. In their study, the *b* axis of the close-packed layer (see Fig. 3 ▸ *c*) was put on the stretching direction while, in our study, the *b* axis is put perpendicular to the stretching direction.

### 3.2. Deformation mechanism in early stage below strain of 35%   

**Figure 4:**
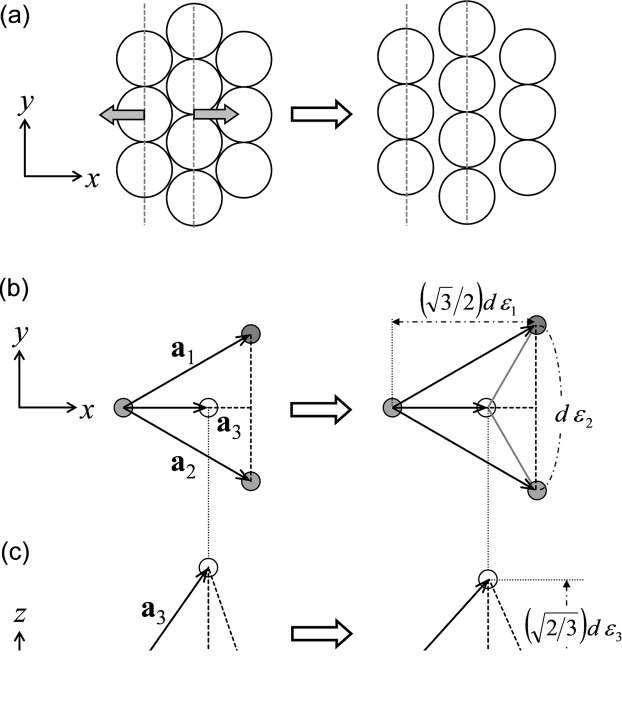
Deformation mechanisms from 0 to 25% strain, presented schematically. (*a*) The motion of particles on the through plane: the inter-sphere distance in the stretching direction increases gradually during uniaxial stretching, while that in the *b* axis does not change. (*b*) A through view of the lattice vectors, **a**_1_, **a**_2_ and **a**_3_, at 0% strain (left-hand side) and at arbitrary strain (right-hand side). The local strains, ∊_1_, ∊_2_ and ∊_3_, are indicated. Gray and white balls represent the center positions of a particle in the lower layer (*A* layer) and in the upper layer (*B* layer), respectively. (*c*) An edge view of the lattice vectors.

A probable model based on a deformed f.c.c. lattice is now proposed for the present results. When the sample is subjected to strain below 35%, particles are gradually separated in the uniaxial stretching direction (**a** + **b**/2 direction) and maintain their distance between neighboring spheres on the *b* axis (Fig. 4 ▸ *a*, particle arrangement). A similar deformation process was observed in a uniaxially stretched SPNP film by transmission electron micrograph observation around crazing points formed during stretching (Choi *et al.*, 2010 ▸). In this model, the cubic symmetry is broken during the stretching process. To analyze the deformed lattice, a ‘primitive lattice’ is useful. The primitive lattice of f.c.c. contains one particle per lattice and the lattice vectors connect the nearest-neighbor particles, as presented in Figs. 4 ▸(*b*) and 4 ▸(*c*).

**Figure 5:**
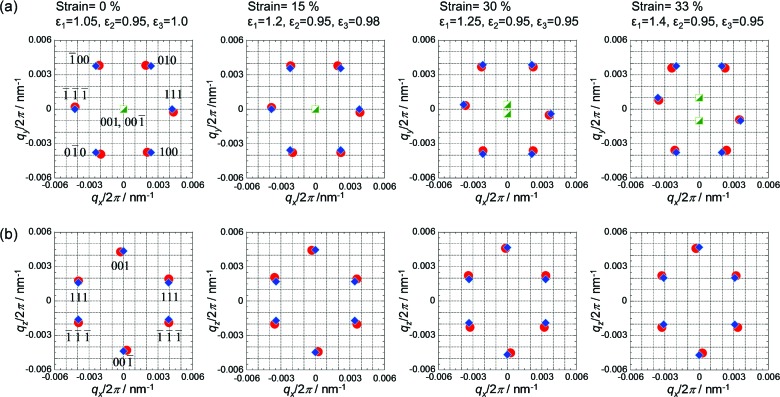
The observed diffraction positions (red circles) and calculated positions (blue diamonds) in (*a*) the through plane and (*b*) the edge plane. The *hkl* indices of the primitive lattice are indicated near the diffraction positions of the far-left patterns. In the upper patterns, the *y* components of the 001 and 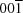 diffractions are projected onto the *xy* plane (half-filled green squares).

**Figure 6:**
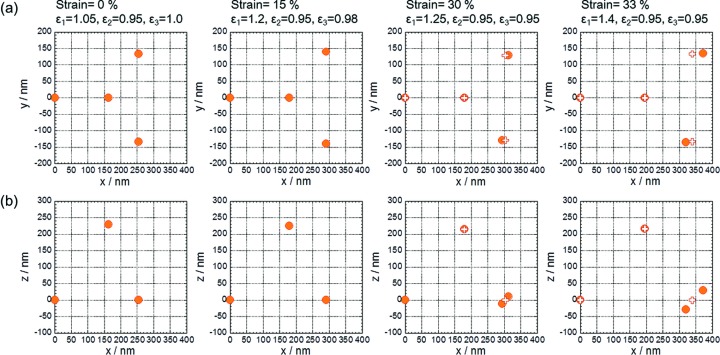
The relative positions of the three neighboring particles to a reference particle at the origin; projection views on (*a*) the *xy* plane and (*b*) the *xz* plane. Until the strain reaches 25%, the positions are calculated from equation (1), while beyond 30% strain, the positions are calculated from the observed diffraction positions. The calculated positions from equation (1) are also plotted in the views at 30% and 33% strain (open ‘+’ symbols).

The lattice vectors are defined as **a**_1_, **a**_2_ and **a**_3_, and the vectors are changed with the strain (Figs. 4 ▸ *b* and 4 ▸ *c*). Vectors **a**_1_ and **a**_2_ lie on the close-packed layer in the through plane and are symmetric across the *x* axis, while vector **a**_3_ is projected out of the through plane. Three local strains in the *x*, *y* and *z* directions are defined as ∊_1_, ∊_2_ and ∊_3_, respectively, which are independent of each other. The deformed lattice vectors in real space are represented by ∊_1_, ∊_2_ and ∊_3_ in the following equations, where *d* is the diameter of the particle
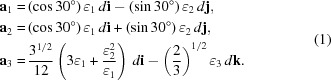
The corresponding reciprocal lattice vectors, **a**_1_^*^, **a**_2_^*^ and **a**_3_^*^, are
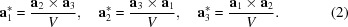
In equation (1), **i**, **j** and **k** are base vectors along the *x*, *y* and *z* axes, respectively. *V* is the volume of the primitive lattice, equal to **a**_1_·(**a**_2_×**a**_3_) (Warren, 1990 ▸). The position of an *hkl* diffraction of the primitive lattice, *q_hkl_*, is represented by equation (3)

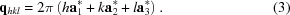
The observed diffractions in Fig. 2 ▸ and the corresponding calculated diffractions at strains of 0, 15, 30 and 33% are presented in the through plane in Fig. 5 ▸(*a*) and in the edge plane in Fig. 5 ▸(*b*). The macroscopic strain and local strain (∊_1_, ∊_2_, ∊_3_) are indicated above the patterns. Hereinafter, *hkl* indices without square brackets [] represent those of the primitive lattice. In the through USAXS patterns, the 111 and 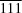 diffractions appeared on the equator as in Fig. 5 ▸(*a*). At a larger strain near 30%, the 111 and 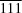 diffractions in the through plane gradually moved off the equator downwards and upwards, respectively. (The asymmetric geometry of the through pattern implies that X-rays through the *z* axis irradiated a single-crystal-like grain rather than the twinned-crystal domain. It should be noted that the path lengths of the X-ray beam are different between the *z* and *y* directions. In the former case, it was at most 1 mm, while in the latter case it was larger than 4 mm. Therefore, the single-crystal-like pattern was observed in the through pattern, while the twinned-crystal-like pattern was observed in the edge pattern). On the other hand, the 010 and 100 diffractions and 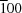 and 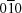 diffractions in the through plane kept the symmetry across the *x* axis. In the edge USAXS patterns, the 001 and 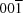 diffractions on the meridian gradually shifted to the high-*q* region along the *q*_*z*_ axis up to 35% strain. These results regarding the 111 and 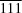 diffractions on the through plane and the 001 and 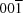 diffractions on the edge plane indicate that the lattice vector **a**_3_^*^ lay on the *xz* plane (edge plane) before the strain reached 30%, and subsequently moved off the *xz* plane at a strain greater than 30% (the projected positions of these diffractions are plotted in Fig. 5 ▸ *a* as half-filled squares). By the gradual increase in the *q_y_* component in the **a**_3_* vector, the 001 and 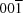 reciprocal-lattice points are gradually separated from the surface of the Ewald sphere (the *xz* plane), resulting in weakening of the 001 and 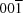 diffractions. The behavior of the 001 and 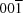 diffractions is consistent with that of the 111 and 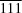 diffractions on the through pattern. In real space, the emerging *q*_*y*_ component of **a**_3_* corresponds to symmetry breaking of **a**_1_ and **a**_2_ across the *x* axis at a larger strain around 30%. In other words, the ‘line-like’ arrangement of particles along the *y* axis (indicated by dashed lines in Fig. 4 ▸ *a*) was broken and the relation of equation (1) was not satisfied at strains greater than 30%. The motion of the particles in the above discussion is presented by projection views in Fig. 6 ▸, where the relative positions of three neighboring particles to a reference particle at variable strains are projected on the *xy* and the *xz* planes. The reference particle is placed at the origin. The relative positions at 30% and 33% are calculated from the observed diffractions, supposing that the 001 and 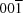 reciprocal vectors have a non-zero *q_y_* component as drawn in Fig. 5 ▸(*a*), while those at 0% and 15% are calculated by equation (1).

### 3.3. Deformation mechanism in late stage above strain of 35%   

**Figure 7:**
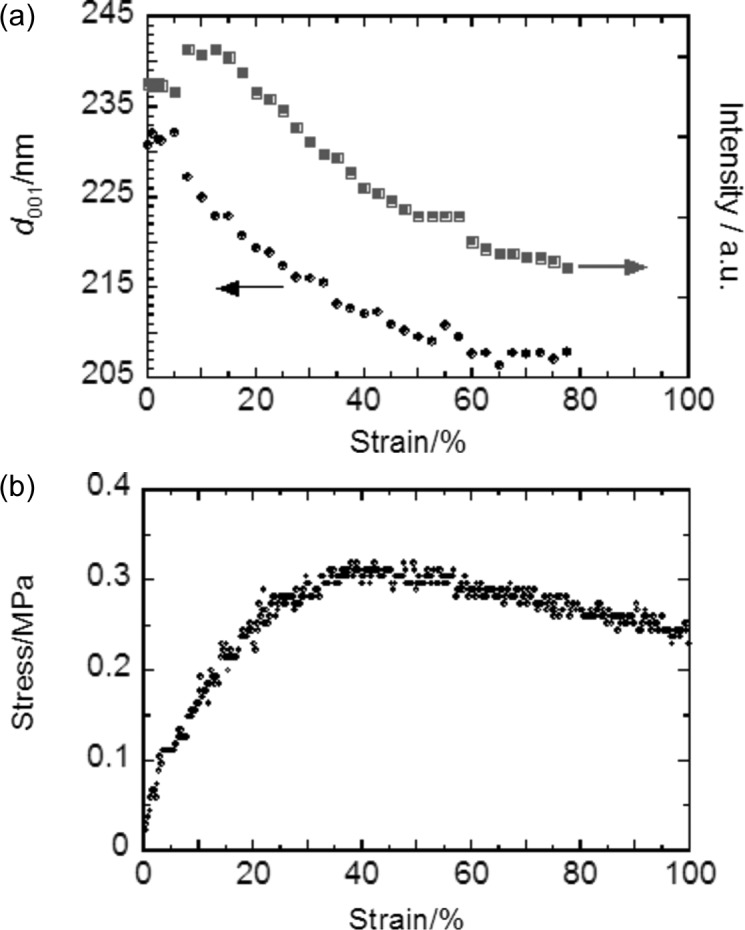
(*a*) The strain dependence of the *d* spacing and intensity of the diffractions appearing on the meridian in the edge pattern. The diffractions are indexed to 001 and 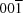 of the primitive lattice in the region of strain below 35%. (*b*) The corresponding stress–strain curve (SS-curve) of the SPNP film during uniaxial stretching at 10% min^−1^.

After the initial stage, the 111 and 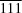 diffractions in the through pattern gradually disappeared and a four-point diffraction pattern was observed at a strain of 43%. Then, diffractions on the equator gradually appeared again in the through plane. In the edge pattern, the 001 and 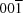 diffractions on the meridian had weakened significantly by a strain of 40% and finally disappeared at 98%. The spacing of these diffractions, *d*_001_ and 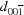, decreased continuously to approximately 90% of the initial value during the early stage (strains of 0 to 35%) and became nearly constant at strains above 40%. The *d* spacings and intensities of these diffractions are plotted with strain in Fig. 7 ▸(*a*). These behaviors of the 001 and 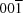 diffractions suggest that the distorted f.c.c. lattices remained but did not contribute to the distortion and gradually disappeared after the strain exceeded 35%.

**Figure 8:**
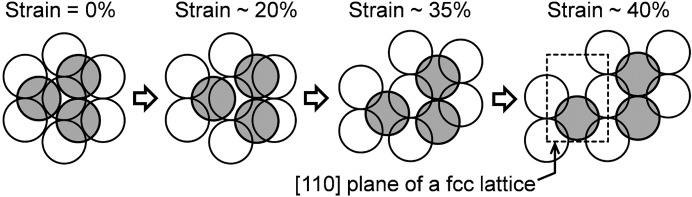
The rearrangement of the nanoparticles in the through plane during uniaxial stretching, illustrated at each strain. White and gray balls represent nanoparticles in the lower (*A*) and upper (*B*) layers, respectively. The [110] plane of the f.c.c. lattice is indicated by a dashed square.

It can be concluded that the weak 001 and 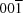 diffractions on the meridian of the edge pattern were independent of the other diffractions and the edge pattern is essentially the same as a four-point diffraction pattern. The discontinuous change from a distorted hexagonal geometry to a four-point geometry in the through and edge patterns implies that the particle arrangement at a strain of 43% is no longer represented by the distortion of the initial f.c.c. lattice. This complex structural change is presumably explained by the formation of another f.c.c. lattice with a different orientation. When the particles on the dashed line in Fig. 4 ▸(*a*) mutually slide in the *y* direction and the distance between the particles in the *x* direction reaches ∼2^1/2^ *d* (*d* is the diameter of the particle at 0% strain), a new [110] plane of an f.c.c. lattice appears in the through plane. The four-point diffraction pattern observed at a strain of 43% is well accounted for by an f.c.c. crystal with the [110] plane perpendicular to the incident X-ray beam. Subsequently, the particles in the upper layer are gradually inserted into the empty space formed in the *A* layer and new [111] planes are formed. The motion of the particles on the *xy* plane is shown schematically in Fig. 8 ▸.

Finally, a quasi-hexagonal pattern is observed at a strain of 98%, similar to the initial hexagonal pattern. The through and edge patterns at 98% are apparently similar to the edge and through patterns at the initial state (0%), respectively. This result implies that the SPNPs form another quasi-f.c.c. crystal where the close-packed layers are nearly parallel to the edge plane. Presumably, the close-packed layers are formed in a rearrangement and rotation of the lattice above 40% strain. The diffractions appearing at the equator of the edge pattern at 98% may correspond to 111 and 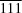 diffractions of the new f.c.c. lattice.

### 3.4. Relationship between f.c.c. crystal orientation and selective reflection   

**Figure 9:**
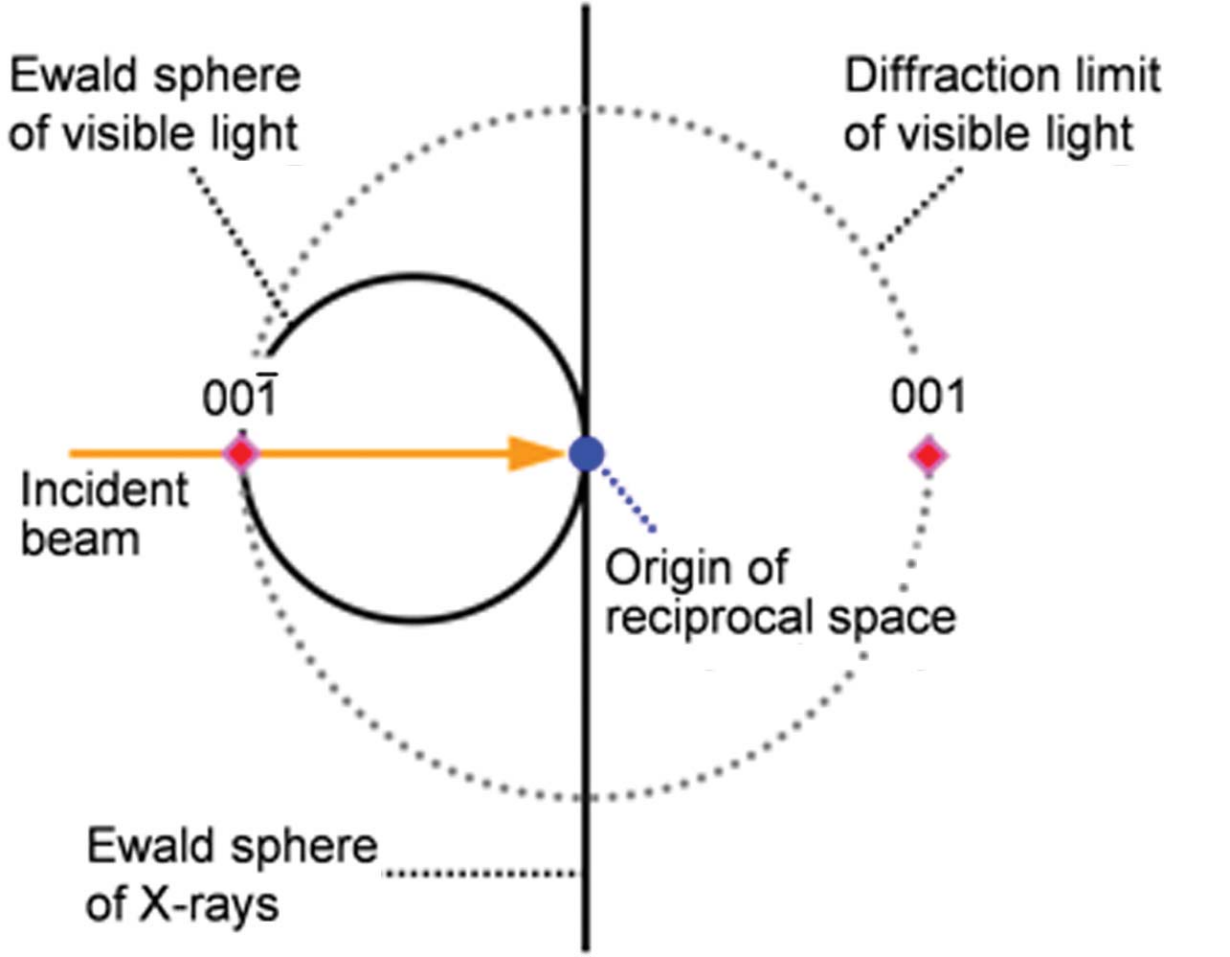
A schematic illustration of the Ewald spheres of visible light and X-rays (solid lines).

The strain dependence of *d*_001_ and 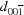 was well consistent with the change in selective reflection color (structural color) observed for incident light on the *z* axis. These values decreased from 229 to 206 nm in the early stage (strains from 0 to 35%) and became nearly constant (Fig. 7 ▸ *a*). In visible-light reflection spectra of the molded SPNP film during uniaxial stretching, the reflection peak shifted to shorter wavelengths in the initial stage, and the peak wavelength became constant and the reflection intensity declined after the strain reached 35% (the details were provided in Fig. 4 of our previous paper; Williams *et al.*, 2015 ▸). In the vertical reflection condition, supposing that the refractive index, *n*, of the matrix polymer is 1.5, the wavelength of the selective reflection at a strain of 0% is evaluated at 687 nm, which is well consistent with the peak wavelength, λ_vis_, in the reflection spectra (Williams *et al.*, 2015 ▸). The Ewald sphere of visible light (the radii are *n*/λ_vis_ < 0.005) is 100 times smaller than that of X-rays (∼1), and the Ewald sphere of X-rays approximates a wall compared with that of visible light, as seen in Fig. 9 ▸. In this case, only the 001 and 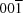 reciprocal lattice points, which are the closest to the origin, satisfy the Bragg condition for visible light in the vertical incidence condition and the other points are out of the diffraction limit of visible light (dotted circle in Fig. 9 ▸). Therefore, the observed structural color is assigned to a selective reflection from the close-packed layer in the through plane (001 and 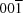 planes of the primitive lattice).

### 3.5. Relationship of structural change with stress–strain behavior   

The mechanical behavior of the molded film also corresponded well to the local structural change observed by USAXS. In the SS-curve in Fig. 7 ▸(*b*), the stress value increased up to a strain of 35% and then gradually decreased up to 100% through a short plateau from 35 to 45%. The SS-curve suggests that the film is elastically deformed up to 30% and plastically deformed above 30%. The strain value of 35% corresponds well to the end of the initial stage of the deformation. The structural change in local scale is well explained by the distorted f.c.c. lattice below a strain of 35%. This result indicates that the film memorizes the original f.c.c. structure and shows elastic deformation character. On the other hand, the distorted f.c.c. structure gradually disappeared after the early stage. This structural transition induces energy dissipation and plastic deformation. Additionally, the film stretched by up to 30% tended to recover the original length during the unloading process (the residual strain was about 10%), while that stretched up to 98% strain did not recover the length.

## 4. Conclusions   

The change in crystal structure of polymer-grafted nanoparticles during uniaxial stretching was investigated by simultaneous USAXS and stress–strain measurement. Before stretching, the particles formed a twinned f.c.c. crystal structure where the 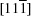 planes are nearly parallel to the *xy* plane. The deformation process could be divided into two regimes, an elastic deformation regime from 0 to 35% of strain, and a plastic deformation regime above 35%.

In the elastic regime, the ordered structure was well modeled by a distorted f.c.c. lattice, and the distance between the 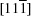 planes, 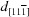, decreased with increasing strain. On the other hand, in the plastic regime, the distorted f.c.c. lattice gradually transformed into another f.c.c. structure and finally vanished at a strain of 98%, while 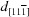 of the remaining distorted f.c.c. lattice kept a constant value. The strain dependence of 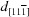 (*d*_001_ of the primitive lattice) was consistent with that of the visible-light reflection. It was evident that the 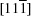 plane selectively reflected visible light.

The structural change in the plastic regime was very complicated. The USAXS patterns inferred that the other f.c.c. structures were newly formed; one was observed at around 43% strain with the [110] plane parallel to the *xy* plane, and another was observed at 98% with the 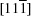 plane parallel to the *xz* plane. The former structure gives four-point diffractions in both the through and edge patterns, while the latter gives a hexagonal pattern in the edge view. In such a structural transition, the ratio of |**a**_1_|/|**a**_1_ − **a**_2_| in Fig. 4 ▸ gradually changes from 1/1 to 1/2^1/2^ and finally 1/3^1/2^.

The deformation mechanism will be accounted for by the character of the grafted polymer. During the stretching process, the lattice parameter |**b**| scarcely decreased with strain, while |**a**| increased with strain (Figs. 3 ▸ and 5 ▸). This means that the polymer-grafted particles have a hard-sphere character, even though the particles have soft shell (layer) of grafted polymer. A grafted polymer with a degree of polymerization of 310 is expected to be in a concentrated polymer-brush (CPB) regime or, at least, a boundary regime between CPB and the semi-dilute polymer-brush (SDPB) regime (Ohno *et al.*, 2007 ▸; Williams *et al.*, 2015 ▸; Choi *et al.*, 2013 ▸). The ends of the grafted polymers on neighboring particles are considered to touch each other at the top surface of the CPB shell and to be physically crosslinked by hydrogen bonds. It is difficult for the polymer chains in the CPB shell of one particle to penetrate into the CPB shell of another particle, due to the high osmotic pressure of a CPB. On the other hand, the polymer chains are allowed to extend in a direction away from the core. The CPB characteristics inhibit the particles from approaching more than in the original state and allow the particles to separate each other. The hydrogen bonds are not considered to bring neighboring particles closer together because the graft polymers can not penetrate into the CPB shell. Consequently, the SPNPs can only be contracted in the direction perpendicular to the close-packed layer and the stretching direction. This behavior is in contrast with ordinary polymer rubbers, which contract in any direction perpendicular to the stretching direction, keeping the volume constant. Therefore, the CPB character of the shell is essential for effective control of the distance between the close-packed layers and also of the structural color of SPNP colloidal crystals by mechanical deformation.
